# Analysis for Patient Survival after Open Abdomen for Torso Trauma and the Impact of Achieving Primary Fascial Closure: A Single-Center Experience

**DOI:** 10.1038/s41598-018-24482-0

**Published:** 2018-04-18

**Authors:** Yu-Pao Hsu, Yon-Cheong Wong, Chih-Yuan Fu, Shang-Yu Wang, Chien-Hung Liao, Chun-Hsiang Ou Yang, Kuo-Ching Yuan

**Affiliations:** 1 Division of Trauma and Emergency Surgery, Department of Surgery, Chang-Gung Memorial Hospital, Linkou, Taiwan; 2 Division of Emergency and Critical Care Radiology, Department of Medical Imaging and Intervention, Chang-Gung Memorial Hospital, Linkou, Taiwan; 3 Department of Emergency and Critical Care Medicine, Division of Acute Care Surgery and Trauma, Department of Surgery, Taipei Medical University Hospital, Taipei, Taiwan

## Abstract

Open abdomen indicates the abdominal fascia is unclosed to abbreviate surgery and to reduce physiological stress. However, complications and difficulties in patient care are often encountered after operation. During May 2008 to March 2013, we performed a prospective protocol-directed observation study regarding open abdomen use in trauma patients. Bogota bag is the temporary abdomen closure initially but negative pressure dressing is used later. A goal-directed ICU care is applied and primary fascial closure is the primary endpoint. There were 242 patients received laparotomy after torso trauma and 84 (34.7%) had open abdomen. Twenty patients soon died within one day and were excluded. Among the included 64 patients, there were 49 (76.6%) males and the mean Injury Severity Score was 31.7. Uncontrolled bleeding was the major indication for open abdomen (64.1%) and the average duration of open abdomen was about 4.2 ± 2.2 days. After treatment, 53(82.8%) had primary fascia closure, which is significant for patient survival (odds ratio 21.6; 95% confidence interval: 3.27–142, p = 0.0014). Factors related to failed primary fascia closure are profound shock during operation, high Sequential Organ Failure Assessment Score in ICU and inadequate urine amount at first 48 hours admission.

## Introduction

Open abdomen (OA) indicates a specific surgical technique in which the abdominal fascial edges are intentionally left unapproximated after laparotomy. It allows a patient to return to the Intensive Care Unit (ICU) earlier from the operation theater under metabolic derangements and facilitates the performance of further definite procedures^1^. The benefits of OA include a shorter operation time in unstable patients, fewer postoperative complications, and the prevention and mitigation of lethal early multiple-organ failure^2^. In addition to being used for abdominal trauma, OA is now part of the Damage-Control Surgical (DCS) strategy for various complicated abdominal conditions, such as severe abdominal sepsis, necrotizing pancreatitis, abdominal compartment syndrome (ACS), necrotizing fasciitis of the abdominal wall, and uncontrolled bleeding in physiologically exhausted patients^3^. Moreover, ACS is often encountered after massive fluid resuscitation and blood transfusion in trauma patients and is now considered a decisive factor contributing to mortality^4^. With the improved understanding of multiple-organ failure in trauma and ACS, damage-control laparotomy with OA is now an important surgical strategy used in traumatology to provide the best results. Although the actual percentage of OA application in trauma patients is not clear, this approach is now widely applied.

However, many challenges encountered in OA patient care. Morbidity and potential complications resulting from OA are increasingly observed. Caring problems frequently happens including difficulties fluid balance maintenance, risk of enterocutaneous fistula, fascial retraction with loss of the abdominal domain, and significant protein loss within the ascites^5^. Although there are various surgical techniques and non-anatomical coverage options for restoring the abdominal domain, the ideal outcome remains to restore the normal anatomic architecture of the abdominal wall using primary fascial closure (PFC). However, some patients may experience failed PFC^1,6–8^, which usually leads to prolonged hospitalization and less favorable outcomes. But risk factors associated with failed PFC and the impact of failed PFC on patient survival after OA in major abdominal trauma are not well elucidated.

The purpose of our study was to review the use of OA after major abdominal trauma in patients in a level-one trauma center in Taiwan. We sought to identify the independent risk factors associated with PFC and the impact of failed PFC on patient outcomes.

## Results

### Demographic data

**Figure 1:**
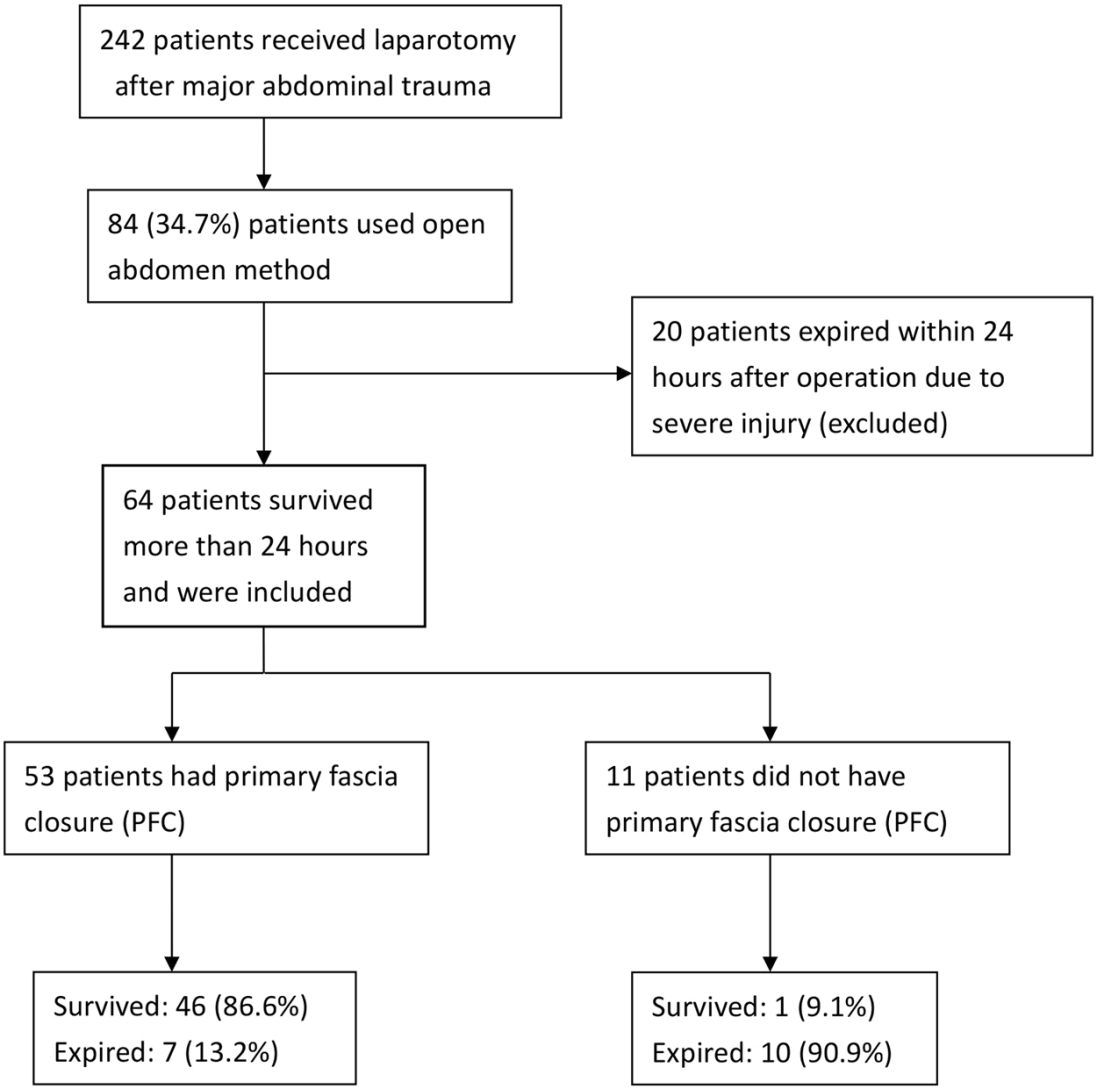
Flowchart of patient enrollment.

From May 2008 to March 2013, 2,949 patients were admitted to the Department of Trauma and Emergent Surgery at Chung Gung Memorial Hospital, Linkou, Taiwan. Among them, 242 (8.2%) patients underwent damage control laparotomy, and OA was used in 84 patients. However, 20 patients died within 24 hours after the operation and were excluded from analysis (Fig. 1). There were 49 men and 15 women included for analysis (Table 1) and the mean ISS was 31.7. Twenty eight (43.8%) patients presented with shock at the triage. Most had severe abdominal injury, and the mean abdominal anatomical injury score (AIS) was 5.7 ± 2.6. The indications for OA were uncontrolled bleeding (64.1%), retroperitoneal hematoma (39.1%), profound shock (31.3%), and severe tissue edema (25%). After treatment, 53 (82.8%) patients achieved PFC. The average number of laparotomies was 2.4 (range, 1–6), and the average duration of OA was 4.2 ± 2.2 days. There were 17 patients died after treatment, so the mortality rate was 26.6%.

**Table 1:** Demographic data, injury severity and treatment result.

	Patient, N = 64	%
Demographic data		
Age	35.8 ± 17.2	—
Sex		
Female	15	23.4%
Male	49	76.6%
ISS	31.7 ± 13.3	—
Abdominal total AIS	5.7 ± 2.6	—
Trauma Mechanism		
Falling	7	10.9%
Heavy compression	5	7.8%
MBA^1^	37	57.8%
MVA^2^	7	10.9%
Pedestrian accident	5	7.8%
Penetrating injury	3	4.7%
ED presentation		
Shock at ED	28	43.8%
Metabolic acidosis in ED	20	31.3%
Coagulopathy at ED	35	54.7%
ED transfusion (Unit)	12.8 ± 19.4	—
OR		
retroperitoneal hematoma	25	39.1%
tissue edema	16	25.0%
profound shock	20	31.3%
uncontrolled bleeding	41	64.1%
OR BLOOD LOSS (ml)	2,955.5 ± 2,379.4	—
ICU		
Initial SOFA SCORE	7.3 ± 3.8	—
First 48 hours balance (ml)	3,521.7 ± 4,751.8	—
Initial 48 hours urine (ml/kg/hr)	1.3 ± 0.8	—
Treatment result		
LOHS^3^ (days)	27.2 ± 22.2	—
Duration of open abdomen (days)	4.2 ± 2.2 (1–11)	—
Primary fascia closure (PFC)	53	82.8%
Mortality	17	26.6%

^1^Motorbike accident, ^2^Motor vehicle accident ^3^Length of hospital stay.

### Clinical presentations

In the analysis of demographics and emergency department presentations between survivors and non-survivors (Table 2), sex, age, and ISS were similar. Non-survivors were more likely to present to the emergency department with head acidosis and shock, but not statistically significantly so. Patients who died presented to the emergency department with significantly higher RTSs and more coagulopathy. In the operation room, the non-survivors were noted to have more FFP use indicating possible more profound shock during operation. We found that the attainment of PFC was significantly associated with patient survival (Table 2). Additionally, we also noted that SOFA score at ICU admission, plasma transfusion during the operation, and number of inotropic agents used after the operation were also statistically significant for survival. The total amount of blood transfused during the first 48 hours had borderline significance (p = 0.05). In the multivariate analysis, the achievement of PFC, plasma transfused during operation, and the SOFA score were significant for patient survival (Table 3).

**Table 2:** Outcome factors analysis after open abdomen.

	Alive, n = 47	Expired, n = 17	p
Demographic data			
Age	35.1 ± 16.2	37.5 ± 19.9	0.625
Sex (M/F)	36/11	13/4	0.992
ISS	31.1 ± 12.5	33.3 ± 15.6	0.558
Abdomen AIS	5.8 ± 2.4	5.6 ± 3.2	0.810
RTS^1^	6.1 ± 1.7	4.4 ± 2.4	0.003
Head injury	9 (19.1%)	5 (29.4%)	0.380
**OA factor**			
Duration of open abdomen(days)	4.22 ± 2.4	4.02 ± 1.1	0.831
NO. of laparotomy	2.4 ± 0.8	2.3 ± 0.9	0.462
Achieve PFC	46 (97.9%)	7 (41.2%)	0.000
ED			
EDABG^2^-pH	7.27 ± 0.13	7.08 ± 0.24	0.008
EDABG-HCO_3_	19.54 ± 4.16	14.77 ± 3.57	0.000
ED Hemoglobin	10.4 ± 2.7	9.3 ± 3.1	0.194
ED Platelet	155.3 ± 89.11	128.76 ± 85.66	0.292
ED INR	1.86 ± 1.73	2.2 ± 1.01	0.450
Coagulopathy at ED	28 (59.6%)	15 (88.2%)	0.031
OR			
PRBC (unit)	8.7 ± 6.9	14 ± 13.9	0.150
FFP (unit)	7.1 ± 6.0	12.5 ± 9.4	0.009
Blood loss (ml)	2578.7 ± 2122.2	3997.1 ± 2788.3	0.420
ICU			
SOFA SCORE at ICU admission	5.9 ± 3.0	10.9 ± 3.4	0.000
NO. of inotropic use in ICU	0.4 ± 0.68	1.35 ± 0.7	0.000
First 48 blood transfusion (ml)	3890.19 ± 3867.59	7416.24 ± 6685.18	0.053
First 48 hours urine (ml/kg/ hour)	1.47 ± 0.62	0.99 ± 0.99	0.037
First 48 hours balance (ml)	2530.77 ± 2857.70	6261.24 ± 7387.22	0.058

^1^Revised trauma score.

^2^Arterial blood gas in ED.

**Table 3:** Multivariate analysis for survival after open abdomen.

Variables	OR (95% CI)	p
Primary fascia closure	21.6 (3.27–142)	0.0014
OR^1^_FFP	0.946 (0.902–0.992)	0.021
SOFA^2^ score	0.799 (0.731–0.874)	<0.001

^1^Operation Room.

^2^Sequential Organ Failure Assessment (SOFA) Score.

### Primary Fascia Closure

A subanalysis for factors related to PFC was performed. Significant predictors of PFC achievement included lower initial SOFA score at ICU admission, lower IVF amount infused during the first 48 hours after admission, and a greater urine output in the first 48 hours after ICU admission. These favorable factors relating to successful PFC actually indicates good physiological response to our treatment. OA, as part of the damage control laparotomy, is to mitigate the physiological dearrangement after traumatic shock and PFC is feasible only in those who can recover well. Therefore, a good recover of patient is the core determination for successful PFC. Moreover, patients who received OA due to profound shock during the operation or who presented to the emergency department with metabolic acidosis also had significantly unfavorable PFC results. In the multivariate analysis, the significant factors associated with failed PFC included profound shock during the operation, high SOFA score at ICU admission, and low urine output during the initial 48 hours after ICU admission (Table 4).

**Table 4:** Multivariate analysis for failed Primary Fascia Closure (PFC).

Variable	OR (95% CI)	p
Profound shock during operation	4.78 (1.47–15.5)	0.0092
High SOFA SCORE	1.35 (1.1–1.67)	0.0042
Low First 48H urine (ml/kg/hr)	1.96 (1.47–2.63)	<0.001

## Discussion

Since the 1990s, the use of OA has gradually increased as an important part of damage-control surgery (DCS) for severe torso trauma^9^. DCS usually includes sequential surgeries after initial resuscitation^10^. It is a well-accepted strategy in modern trauma management because it avoids extensive procedures on unstable patients and emphasizes stabilization of fatal physiological derangements. The intra-operative indications for DCS/OA include operative findings or fragile physiological conditions^11,12^. It has been reported in the previous study that mortality can be reduced if DCS was decided early^13^. Regarding this study, we identified the indications for OA from the operation record, so the indications were all based on operative findings. Bleeding that was difficult to control was the most common indication, accounting for 64.1% of patients and similar application for DCS were found in literatures. The awareness of uncontrolled bleeding during operation, such as diffuse oozing without a distinct bleeding vessel or lack of clot in a pool of blood, has been described in the literature as an indication for open abdomen to shorten the laparotomy^14^. Exsanguination during laparotomy that resulted in transfusion of more than 10 units packed red blood cells (PRBCs) is also an indication for DCS^9^. Besides, open abdomen is indicated as a prophylactic management in patients who are high risk for postoperative abdominal compartment syndrome.The risk factors reported in literature include more than 15 L of crystalloid infusion; more than 10 units of PRBCs transfusion; increased peak inspiratory pressure more than 40 mmHg during abdomen wound closure^4,11,15,16^. In our study, the mean amount of blood loss for these OA patients was about 3000 ml, or approximately 12U in Taiwan. Our indication for OA is consistent with the indications in literature such as uncontrolled bleeding.

Although the use of DCS with OA is emerging, factors predicting patient outcomes after OA for trauma patients are not well elucidated. Several factors related to patient outcomes after OA were identified in this single-center study. Non-surviving patients usually presented with more severe coagulopathy and a lower RTS at triage. The RTS is based on the Glasgow Coma Scale, SBP, and respiratory rate, all of which are very indicative of the patient’s physiological condition. The RTS measured at the triage is usually performed before resuscitation, and a lower RTS precisely indicated worse physiological conditions in the non-survivals. Coagulopathy is the manifestation of traumatic bleeding. Non-surviving OA patients possibly sustained more severe bleeding and therefore more severe coagulopathy. Although non-surviving patients had a higher ratio of head injury; head injury alone is not considered significant for OA patient survival. We also found the difference in abdominal AIS was not significant between the surviving and non-surviving patients, suggesting that the anatomical injury pattern may be similar between them but the physiological indicators can tell apart. Shock status (SBP < 90 mmHg) and acidosis status (pH < 7.2) at the triage were both more severe for non-surviving patients, but neither was significant, possibly due to such compensatory mechanisms as tachycardia and vasoconstriction. Because of lacking immediate compensatory mechanism for coagulation, the severity of coagulopathy correlated with bleeding and resulted in significant different.

The amount of fresh frozen plasma transfused intraoperatively and the total amount of blood transfused during the initial 48 hours after admission were both important for patient survival in our study. The amount of plasma transfused is considered a quantitative indicator of resuscitation. A ratio of ~1:1 between PRBCs and plasma is advocated as an effective strategy in massive transfusion for trauma patients. Therefore, plasma transfusion is now widely used in trauma resuscitation. The SOFA score measured at ICU admission and the number of inotropic agents used after the operation were both significant for patient survival in this study (Table 2). The SOFA score and the number of inotropic agents used can be regarded as patient responses to initial resuscitation and hemostasis procedures. The SOFA score is a global evaluation of patient physiological conditions, and it, similar to the RTS, clearly describes physiological differences between surviving and non-surviving patients. Higher SOFA score is considered to be associated with higher mortality for critical patients, and trauma patients are no different. Even if hemostasis were achieved, the physiological injury caused by hemorrhagic shock can be so severe that hemodynamic status remains unstable due to low systemic vascular resistance. For this situation, inotropic agents are indicated to stabilize patients, and the use of more kinds of inotropic agents is indicative of a more unstable condition. After resuscitation and the operation, it is crucial to evaluate precisely the effectiveness of resuscitation, and urine output is considered a simple and representative indicator of tissue perfusion. In our study, urine output in the first 48 hours after ICU admission also predicted patient outcomes. In the multivariate analysis, achievement of PFC, plasma transfusion, and initial SOFA score were further confirmed to be significant for OA patient survival (Table 3).

As successful PFC is critical for patient survival, factors associated with PFC achievement are worthy of discussion. Studies have advocated that early PFC provides better patient outcomes by facilitating the restoration of the abdominal domain and mitigating morbidity associated with prolonged OA^2^. There are two types of OA patients. Regarding the uncomplicated type patient, the OA is expected to be closed no more than 4–7 days. A high rate of PFC can be achieved, and these patients often recover well not related to the choice of TAC^17–19^. The second type is complicated OA patients requiring more resuscitation procedures and having bumpy hospital courses. The duration of open abdomen in these patients is usually beyond 7 days; can be up to 20–40 days^6,7,20^. The significant factors reported in the literature that related to the complicated course include prolonged OA duration; multisystem injuries involving colon or duodenal injury; and severe infections^18,21^. Although all of the OA patients in our study sustained severe and multiple trauma with mean ISS about 31.7 ± 13.3 (Table 1), we still achieved early PFC as possible with the mean duration of open abdomen about 4.2 days. There are studies revealing that conservative fluid resuscitation less than 20 L; fewer transfusions; and maintaining a net negative fluid balance, resulted in a better rate of PFC^18,19,21^. Stone *et al*. also reported that OA patients with profound tissue hypoperfusion and failed lactate clearance were less likely to achieve PFC^19^.

In a large-scale study of OA for trauma^2^, some factors associated with failed PFC were revealed: number of laparotomies, intra-abdominal abscess, bloodstream infection, acute renal failure, enteric fistula, and ISS >15. Except for the ISS, most of these negative factors are actually late complications. In our study, we also found some significant factors associated with failed PFC. The negatively associated factors were acidosis status at emergency department presentation, higher SOFA score at ICU admission, less intravenous fluids administered during the initial 48 hours after admission, less urine output during the initial 48 hours after admission, and patients whose indication for OA was profound shock. Acidosis status at emergency department presentation and profound shock during the operation are considered physiological indicators of hypoperfusion. PFC failure among these patients is likely due to a higher mortality rate after severe torso trauma for those patients. Although ISS was considered important for PFC in a study^2^, our study showed more significant results for physiological parameters, such as acidosis at emergency department presentation and SOFA score at ICU admission. In the multivariate analysis, profound shock, high SOFA score, and inadequate urine output during the initial 48 hours after ICU admission significantly predicted failed PFC (Table 4).

There are many approaches for TAC after OA. The best and most appropriate TAC is the one that maintains abdominal viscera in a physiological environment; causing little damage to the fascia and protects underlying bowel from injury. The TAC also prevents contamination; drains out peritoneal fluid, avoids adhesions, and relieves ACS. Another important demand for TAC is to decrease abdominal wall retraction preparing for later PFC^22^. Current options of TAC are classified into three: the skin-only closure techniques; negative pressure dressing and the Bogota bag^15,23^. However, there is a lack of substantial studies comparing the long-term results among these different TACs in traumatic OA, and none has been proven superior for PFC. In our study, we used the Bogota bag made of a sterile intravenous bag as the initial choice of TAC, which is also primarily used in our institute. Oswaldo Borraez invented the Bogota bag in 1984, but Mattox coined the name after visiting with surgeons in Bogota, Colombia, in 1997^24,25^. There are many advantages of using Bogota bags in traumatic OA. First, the bag is easily and rapidly available. The bag can be tailored for each patient in order to provide maximal expansion of the peritoneal cavity. Furthermore, the transparency of the plastic intravenous bag allows direct inspection of intra-abdominal bleeding and bowel perfusion, which is very important for trauma treatment and prompt response. The major shortcomings of the Bogota bag are easy evisceration at the abdominal wall edge, possible abdominal fascia retraction, and cannot provide effective abdominal fluid drainage^24^. Therefore, we always use two Jackson-Pratt drains along with the Bogota bag.

There are several limitations of our study. Although it was a prospective study with a predesigned protocol, adjustments were often made due to unpredictable patient conditions. Our patient number was also small. Many of the patients were referred from other hospitals, and the resuscitation there was not consistent. The decision regarding when to close the abdomen was at the discretion of the surgeon’s evaluation, and may have varied. We only used Bogota bags for temporary abdomen closure, but other methods may be more beneficial for patient outcomes. A more well-designed prospective study in the future is warranted for a more comprehensive understanding of OA use in trauma patients.

## Conclusion

The OA technique is an important component of DCS in traumatology. The most frequently encountered intraoperative indication was uncontrolled bleeding (64.1%) during surgery. The duration of OA for trauma patients was about 2–6 days, and more than 80% of these patients achieved PFC. Failed PFC and worse physiological conditions were significantly associated with patient mortality. In our study, profound shock during the operation, low SOFA score at ICU admission, and inadequate urine output during the first 48 hours after SICU admission predicted failed PFC.

## Methods

### Study design and trauma management

**Figure 2:**
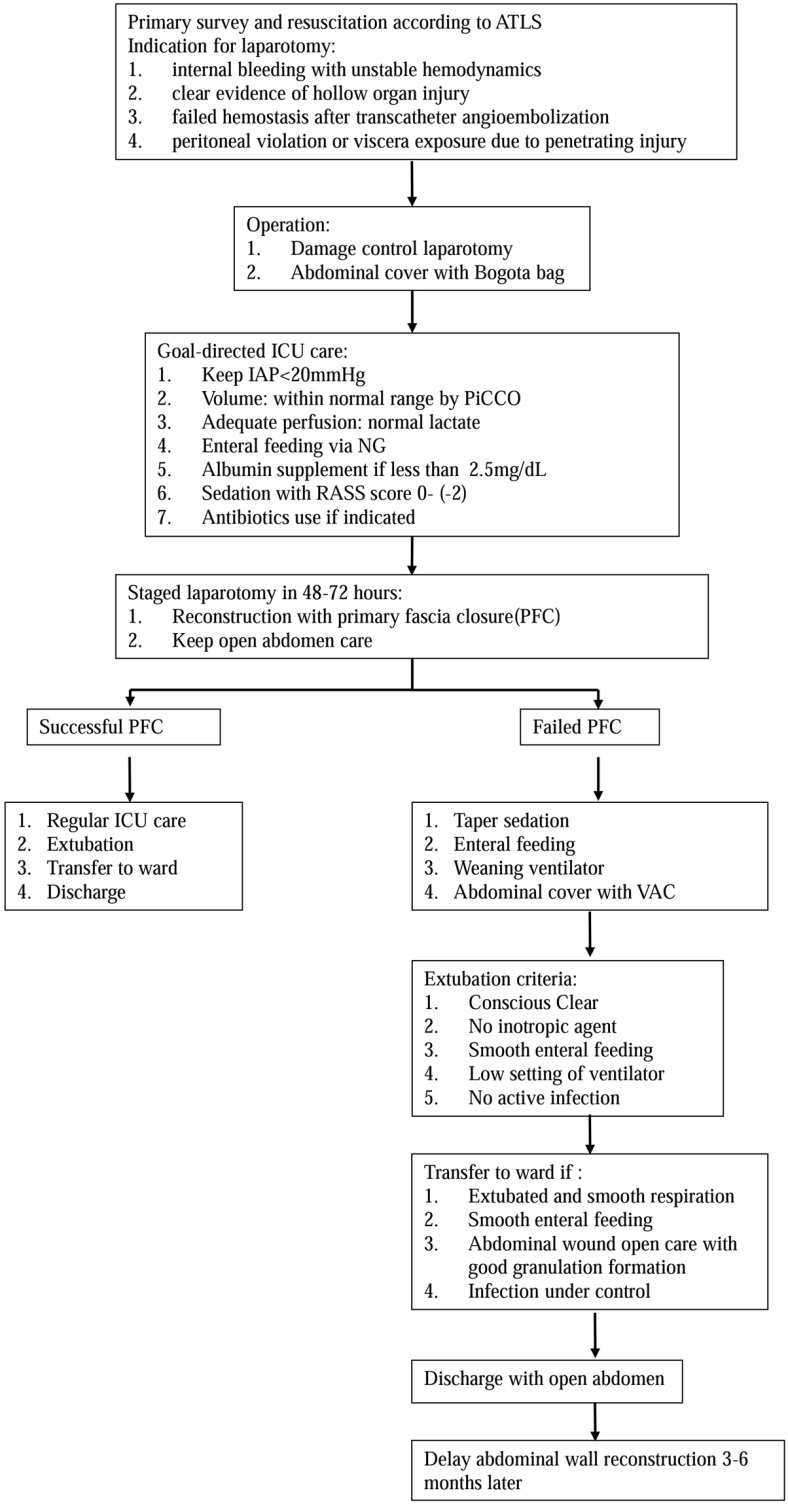
Treatment protocol for traumatic open abdomen.

This is a prospective cohort observational study using a predesigned protocol (Fig. 2). Approval of Chang Gung Memorial Hospital’s Institutional Review Board and a waiver of regulatory informed consent are obtained (Number: 104-7655B). In general, patients sustaining major abdominal trauma received management and resuscitation according to the Advanced Trauma Life Support (ATLS) guidelines in the Emergency Department (ED). Emergent laparotomy was indicated for patients presenting with unstable hemodynamics due to internal bleeding, clear evidence of hollow organ injury on imaging studies, failed hemostasis after transcatheter angioembolization for solid organ injury, or penetrating injury with evidence of peritoneal violation or viscera exposure. During laparotomy, the use of OA was at the discretion of the trauma surgeon according to the operative findings and the patient’s physiological condition. In cases of OA, the Bogota bag was the initial method of temporary abdominal closure (TAC). After the operation, patients were transported to the intensive care unit (ICU) for further care according to the protocol.

### Care for open abdomen patient

Sedation was mandatory for ventilator compliance and for preventing unnecessary abdominal muscle exertion. Further neuromuscular blocking agents were administered in cases of patient-ventilator discrepancy with unstable oxygenation status. Regular hemoglobin level assessments and intra-abdominal pressure measurement were performed for internal bleeding detection. Precise hemodynamic monitoring was achieved with PiCCO^®^ (PULSION Medical Systems SE, Feldkirchen, Germany), and our goal was to maintain volume status within the reference range. Patients without gastrointestinal tract injury received early enteral feeding after inotropic agents were stopped, regardless of OA status. Parenteral nutrition was provided if enteral feeding was not feasible. Antibiotics using third-generation cephalosporin was prescribed as prophylactic use or therapeutic use according to operative findings. Blood transfusion was performed only in cases of low hemoglobin (<8 g/dL), coagulopathy (international normalized ratio of prothrombin time >1.5), or thrombocytopenia (<150,000/µL). Staged operations were arranged within 48–72 hours if patients’ conditions permitted. PFC was the ultimate goal for these patients and was achieved either by a one-stage operation or by multiple operations. If PFC achieved, extubation and standard surgical care was provided for all patients. If PFC failed, patient care was provided by protocol if no contraindications. Negative pressure wound treatment(NPWT) using V.A.C. Therapy (Acelity and KCI Headquarters, 12930 W Interstate 10, San Antonio, TX 78249-2248) was used for TAC and delayed abdominal domain reconstruction was performed 3–6 months later. PFC is the primary endpoint and patient survival, length of hospital stay (LOHS) and complications are our secondary endpoint.

### Statistic analysis

Data obtained included demographic data, trauma mechanisms, injury severity score (ISS), revised trauma score (RTS), associated injuries, operative details, blood transfusion details, sequential organ failure assessment (SOFA) score at SICU admission, intravenous (IV) fluid balance, ventilator record, length of ICU stay, length of hospital stay, and treatment outcomes. Continuous variables were reported as median and quartile and were compared with the nonparametric Mann-Whitney U test. Categorical variables were analysed using the χ^2^ and Fisher’s exact tests. A *p*-value < 0.05 was considered statistically significant and all statistical analysis was done using SPSS^®^ version 20 (IBM, Armonk, New York, USA). Clinically significant variables were entered into a stepwise logistic regression model to identify independent risk factors for patient survival and achievement of PFC.

### Data availability statement

The datasets generated during and/or analysed during the current study are not publicly available due to institutional policy but are available from the corresponding author on reasonable request.
